# Revisiting the use of the EORTC QLQ-STO22 to assess health-related quality of life of patients with gastric cancer: incorporating updated treatment options and cross-cultural perspectives

**DOI:** 10.1007/s10120-024-01492-8

**Published:** 2024-04-26

**Authors:** S. C. Sodergren, A. Hurley-Wallace, V. Vassiliou, B. Alkhaffaf, B. Batsaikhan, A. S. Darlington, T. Fleitas-Kanonnikof, M. G. Guren, M. Honda, Y. W. Kim, S. Kim, M. N. Krishnamurthy, S. Y. Loh, N. S. Turhal, J. Zhou, K. Dennis, R. Krishnatry, M. Terashima, G. Tsironis, T. Yoshikawa, M. Terada, On behalf of the EORTC Quality of Life Group, Loukia Georgiou, Alice Harvey, Makoto Hikage, Hidekazu Hirano, Loli Iglesias, Hiroto Ishiki, Peng Choong Lau, Beatriz Lopez, Elisavet Papageorgiou, Charlotte Rees, Alison Rowsell, Ioannis Stylianou, Takeyuki Wada, Elizabeth Wedge, Wei Jin Wong

**Affiliations:** 1 School of Health Sciences, University of Southampton, Southampton, SO17 1BJ UK; 2 Medical Oncology Department, Bank of Cyprus Oncology Centre, Nicosia, Cyprus; 3 Department of Oesophago-Gastric & Bariatric Surgery, Northern Care Alliance NHS Foundation Trust, Salford, UK; 4 Division of Cancer Sciences, Faculty of Biology, Medicine and Health, School of Medical Sciences, University of Manchester, Manchester, UK; 5 Department of Internal Medicine, Institute of Medical Sciences, Mongolian National University of Medical Sciences, Ulaanbaatar, Mongolia; 6 Medical Oncology Department, Hospital Clinico Universitario de Valencia, Valencia, Spain; 7 Institute of Clinical Medicine, University of Oslo, Oslo, Norway; 8 Department of Oncology, Oslo University Hospital, Oslo, Norway; 9 General South Tohoku Hospital, Koriyama, Japan; 10 Center for Gastric Cancer, National Cancer Center, Goyang-si, Korea; 11 Department of Clinical Pharmacology, Tata Memorial Centre, Tirupati, India; 12 Faculty of Medicine, University of Malaya, Kuala Lumpur, Malaysia; 13 Anadolu Medical Center, Çayırova, Turkey; 14 Department of General Surgery, The First Affiliated Hospital of Soochow University, Suzhou, China; 15 Division of Radiation Oncology, The Ottawa Hospital Cancer Centre, Ottawa, Canada; 16 Department of Radiology, University of Ottawa, Ottawa, Canada; 17 Department of Radiation Oncology, Tata Memorial Centre, Tirupati, India; 18 Division of Gastric Surgery, Shizuoka Cancer Center, Shizuoka, Japan; 19 Department of Gastric Surgery, National Cancer Center Hospital, Tokyo, Japan; 20 Department of International Clinical Development/International Trials Management Section, National Cancer Center Hospital, Tokyo, Japan

**Keywords:** Cross-cultural comparison, EORTC QLQ-STO22, Gastric cancer, Health-related quality of life

## Abstract

**Background:**

The EORTC QLQ-STO22 (QLQ-STO22) is a firmly established and validated measure of health-related quality of life (HRQoL) for people with gastric cancer (GC), developed over two decades ago. Since then there have been dramatic changes in treatment options for GC. Also, East Asian patients were not involved in the development of QLQ-STO22, where GC is most prevalent and the QLQ-STO22 is widely used. A review with appropriate updating of the measure was planned. This study aims to capture HRQoL issues associated with new treatments and the perspectives of patients and health care professionals (HCPs) from different cultural backgrounds, including East Asia.

**Methods:**

A systematic literature review and open-ended interviews were preformed to identify potential new HRQoL issues relating to GC. This was followed by structured interviews where HCPs and patients reviewed the QLQ-STO22 alongside new issues regarding relevance, importance, and acceptability.

**Results:**

The review of 267 publications and interviews with 104 patients and 18 HCPs (48 and 9 from East Asia, respectively) generated a list of 58 new issues. Three of these relating to eating small amounts, flatulence, and neuropathy were recommended for inclusion in an updated version of the QLQ-STO22 and covered by five additional questions.

**Conclusions:**

This study supports the content validity of the QLQ-STO22, suggesting its continued relevance to patients with GC, including those from East Asia. The updated version with additional questions and linguistic changes will enhance its specificity, but further testing is required.

**Supplementary Information:**

The online version contains supplementary material available at 10.1007/s10120-024-01492-8.

## Introduction

According to the GLOBOCAN 2020 database, gastric cancer (GC) is ranked among the top three most common cancers in 19 countries, with an estimated 1.1 million cases and 770,000 deaths annually [1]. Countries within East Asia (Japan, South Korea, North Korea, China, Taiwan, Mongolia, and Macau) have the highest proportion of GC cases (60% of all cases) [1].

The treatment strategy for GC is constantly evolving and becoming more personalized and complex [2, 3]. In the early 2000s, systemic therapy comprised triplet combinations, such as Epirubicin, cisplatin, 5FU (ECF)/Epirubicin, cisplatin, capecitabine, or similar, and was limited for use with locally advanced tumors [4]. Contemporary therapy uses more intense regimens, such as FLOT (5-fluorouracil, leucovorin, oxaliplatin, and docetaxel), for almost all ≥ Stage IB resectable GCs [5]. Furthermore, recent developments in the understanding of the relationship between oncogenesis and genomic alterations and tumor microenvironment have led to the testing of targeted agents and checkpoint inhibitors for selected patients with advanced GC [6–9].

Additionally, surgical procedures have evolved to include laparoscopic and robotic approaches, which are more sophisticated and less invasive compared with traditional open gastrectomy with lymph node dissection. [10, 11].

While tumor response and survival outcomes are the major drivers behind assessments of treatment efficacy and selection, the impact on health-related quality of life (HRQoL) is becoming equally relevant, described as patient experiences in terms of physical and psychosocial side effects and how these affect their functioning across multiple domains of life. Early reviews of HRQoL assessments in GC trials revealed their limited use and a focus on physical functioning from the clinician’s perspective [12]. While the use of generic cancer multi-dimensional patient-reported measures, such as the Functional Assessment of Cancer-General (FACT-G) [13] and the European Organisation for Research and Treatment of Cancer core measure (EORTC QLQ-C30 (QLQ-C30)) [14], allows for a more comprehensive and patient-centric evaluation of the impact of cancer and its treatment, these scales lack sensitivity in terms of the unique aspects of GC and its treatment. The modular approach to HRQoL assessment adopted by the EORTC Quality of Life Group (QLG) involves the development of modules (questionnaires) specific to an area of interest to supplement the core measure (QLQ-C30) [15]. In the context of GC, the EORTC QLQ-STO22 (QLQ-STO22) [16, 17] was developed to measure all issues of relevance and importance to patients with GC. As with all new EORTC QLG modules, the development of the QLQ-STO22 followed four phases: Phase 1 involved the generation of an exhaustive list of HRQoL issues; Phase 2 operationalized the issues into items (questions); Phase 3 pre-tested and refined the draft questionnaire, and Phase 4 tested the psychometric properties in an international validation study. The QLQ-STO22 includes 22 questions covering five scales (dysphagia; pain/discomfort; dietary restrictions; upper gastrointestinal symptoms; specific emotional problems) and three single questions (dry mouth; body image, and hair loss).

The advent of GC specific HRQoL measures such as the QLQ-ST022, available around the time of Kaptein et al.’s review [12], was heralded as having the potential of offering a gold-standard HRQoL assessment in patients with GC and ensuring that HRQoL is fully embedded within outcome assessments for this patient group. Our subsequent review of publications between 2001 and 2021 [18] highlights the increased focus on HRQoL in GC: 267 papers were included in our review compared with 26 studies captured by Kaptein et al. [12]. Out of the 24 measures identified in our review, the EORTC measurement system represented the most favored approach with 60 and 34% of studies using the QLQ-C30 and QLQ-STO22 respectively [18].

The extensive development process of the QLQ-STO22 provided the opportunity for a large group of patients affected by GC to identify the specific issues of relevance and importance to them. However, with the constantly evolving treatment landscape for GC, it could be argued that the QLQ-STO22 needs updating to enhance its sensitivity to side effects not seen with previous treatments. Furthermore, although the QLQ-STO22 is presented as cross-culturally valid [17], health care professionals (HCPs) and patients from countries within East Asia where GC is most prevalent did not contribute to its development.

The need to address whether the QLQ-STO22 is acceptable for use with patients from East Asia or whether adaptations are needed is of paramount importance and will help guide researchers and clinicians in future in their selection of the optimal method for assessing HRQoL in patients with GC. This study aims to identify whether the QLQ-STO22 needs updating to 1) reflect the HRQoL issues of importance to patients treated for GC with current treatment protocols and 2) ensure relevance to patients across different cultures including East Asia.

## Methods

The methods adopted for this study are informed by the EORTC QLG module development guidelines with specific reference to the guidance for updating modules [16]. The study protocol was peer-reviewed by the EORTC QLG. To update the content validity and include an East Asian perspective, the first phase of module development was revisited with HRQoL issues captured from the existing literature and patient and HCP interviews (Phase 1a) and reviewed for relevance and importance (Phase 1b).

### Systematic literature review

A systematic review of publications between January 2001 and January 2021 reporting HRQoL of patients currently treated with or within 12 months post-treatment for GC was performed. For a full overview of the databases and search terms used, as well as the data extraction and analysis processes adopted, see Rowsell et al. [17].

### Patient and HCP interviews (Phase 1a and 1b)

In-depth semi-structured interviews were performed with patients treated with GC within the last 12 months. Ethical and research governance approvals were obtained at each recruitment center in accordance with local requirements, and all patients provided written informed consent. The study was coordinated from the UK, with patients also recruited from China, Cyprus, India, Japan, Malaysia, Mongolia, South Korea, Spain, and Turkey. Recruitment was stratified according to country (East Asia or non-East Asia) and treatment type. The first phase (1a) of issue generation interviews included patients from Cyprus, Japan, Malaysia, Mongolia, and the UK. Interviews opened with a general question asking patients to describe their experiences of having GC and receiving treatment. They were then shown the QLQ-C30 and the QLQ-STO22 to encourage further discussion and identify questions which were particularly relevant and important to them as well as those which they did not recognize as something they had experienced. In addition, they were asked to identify any questions which were confusing, upsetting, or in need of re-wording. Finally, they were asked to talk about any issues which were missing from the questionnaires. Socio-demographic and clinical data were also collected from patients and their medical notes. See online resource 1 for an overview of the interview schedule.

A second set of interviews (1b) asked a separate group of patients and HCPs from China, India, Japan, Mongolia, South Korea, Spain, Turkey, and the UK to share their experiences and review the list of issues generated from the earlier interviews. In addition, patients were asked to review the content of the current QLQ-STO22 and comment on the wording and acceptability of the questions, as well as identifying any further omissions. Participants were also asked for feedback on any “problem” areas identified by patients in the earlier phase of interviews. The interviews also included a rating exercise whereby the QLQ-STO22 questions and new issues were scored for relevance (Yes/No), defined as having been experienced at some time since diagnosis, and for importance (1 = Not at all, 2 = A little, 3 = Quite a bit, 4 = Very much), defined as the extent to which it was troublesome/bothersome. In addition, patients and HCPs were asked to indicate any questions or issues which should not be included in the questionnaire as well as their top 10 priority questions to be included either from the QLQ-STO22 or the new issue list. Socio-demographic characteristics of patients and HCPs as well as clinical data for patients were recorded. Online resource 2 presents the Phase 1b interview schedule.

### Expert review panel

Decisions relating to recommendations and proposed modifications to the QLQ-STO22 were formulated following a meeting of GC clinicians and researchers from the EORTC QLG. Members of EORTC QLG translation team also advised on linguistic changes required based on cross-cultural and language user feedback. In terms of operationalizing new issues into questions to be included in an updated QLQ-STO22, the EORTC QLG item library was consulted.

### Analysis

Qualitative data (patient and HCP comments) were coded and independently reviewed by two researchers (SS and AHW), and question/issue ratings were analyzed in accordance with the EORTC QLG Module Development Guidelines [16], which in turn helped inform recommendations regarding the questions to be modified, removed, or added. For question/new issue relevance, the number (percentage) of participants rating each as relevant was calculated, while for importance ratings, the mean scores were calculated. According to the EORTC QLG recommendations, questions to be included in a module should have a mean relevance of > 1.5 and rated as important by more than 50% participants, with greater emphasis on patients’ ratings.

The new issues generated in Phase 1a, and presented to patients and HCPs in Phase 1b, were considered to have a high priority for inclusion if at least 20% of the patients or 30% of HCPs rated it among the 10 most relevant items. Both qualitative and quantitative data were compared according to patients’ cultural background with particular attention paid to the importance, relevance, and acceptability of the existing questions. Comparisons were confined to descriptive analyses rather than testing for significant differences, given the small sub-group sample sizes.

## Results

### Systematic literature review

A total of 267 eligible papers were extracted for full review and included 24 HRQoL measures and six studies reporting qualitative data. The EORTC measures (QLQ-C30 and QLQ-STO22) were the most frequently used irrespective of country. In 20 studies, bespoke and non-validated measures were used, more commonly in studies from East Asia [16]. HRQoL issues captured were typically a reflection of the content of the measures adopted, with only six qualitative studies offering insight into additional HRQoL experiences of patients. Issues reported include diarrhea, constipation, reflux, abdominal pain, abdominal fullness or bloating, difficulty swallowing, eating restrictions, and weight loss. Psychosocial issues related to these problems include enjoyment of eating, ability to go out and plan activities, worry and distress. Issues relating to the compatibility of some of the Westernized measures (including the QLQ-STO22) within the East Asian cultures were highlighted, e.g., linguistic equivalence of questions asking about eating restrictions and bloating sensations and cultural acceptability of issues relating to family and practical problems, spiritual and religious beliefs, and sexuality. For a full list of issues captured and measures identified in the review, see Rowsell et al. [18].

### Phase 1a patient interviews

#### Sample

Sixty-one patients, including 25 from East Asia (Japan and Mongolia) participated in the first set of interviews to capture patient experiences of using the QLQ-STO22 and elicit new issues. All participants were currently on treatment except for six patients (five from East Asia). The sample was representative of all stages of disease and multiple treatments. None of the patients recruited from East Asia received chemoradiotherapy or FLOT chemotherapy. In addition, laparoscopic and robotic surgeries were utilized only with patients from East Asia. Socio-demographic and clinical characteristics of the patients are presented in online resource 3.

#### Patients experiences of using the QLQ-STO22

Thirty-two patients identified at least one irrelevant question included in the QLQ-STO22; for 11 including hair loss (11 patients) and difficulties eating liquidized or soft foods (10 patients). Eight patients highlighted at least one question as confusing or difficult to answer. Difficulties with understanding the meaning of “belching” was mentioned by two participants (from Malaysia and the UK) and for the participant from Malaysia, this overlapped with the question on acid or bile. Problems with potentially overlapping questions were also identified by a participant in Spain with respect to pain, discomfort, and bloated sensations in the stomach/abdomen. For two participants from Spain, taking a long time to complete a meal was difficult to answer and was not considered to be a useful reflection on their HRQoL. The word “trouble” was questioned by a participant from Japan in the context of eating in front of other people. For another participant from Japan, problems eating foods were difficult to reflect upon as it depended on whether this was during or after a mealtime. There was also uncertainty about what was meant by the “stomach area” by a participant from Japan. Finally, a participant from Malaysia found the question on feeling physically less attractive as difficult to consider as he had not experienced any changes to his appearance and had no “physical disability”. While the majority of participants did not express any discomfort or upset over any of the questions asked, with one participant from Malaysia reporting: “I am old already, I see all the questions as presented reasonably”, three participants (one from Cyprus and two from Japan) mentioned that the question on the impact of GC on perceived physical attractiveness was upsetting: “I am disgusted because asking a question about attractiveness and appearances is too direct for females in Japan”. This participant from Japan felt that the question about hair loss could also trigger negative feelings.

Thirty-one patients identified at least one issue not currently covered by the QLQ-STO22 (Table 1). A list of 26 issues was generated. The most frequently mentioned (by 7 patients) “new” issues were related to neuropathy, followed by having to eat smaller portion sizes and eat more frequently (4 patients). Issues relating to emotional functioning, in particular worries (about dying, the impact of GC on others) were also recommended for inclusion and highlighted as a priority area. Indeed, one participant from Japan felt that the care received focused on addressing physical symptoms and overlooked the emotional needs of patients. Conversely, a participant from Turkey felt that questions about patients’ emotional state might be offensive and should not be included. Four participants felt the need for a question assessing satisfaction with support and information needs. Two participants felt that the list of questions should not be extended: “I feel 22 questions are already so many, I don’t want to add more”.

**Table 1:** Issues captured from Phase 1a interviews which were not currently covered by the QLQ-STO22

Health-related quality of life issue	Number of patients
Neuropathy	7
Meal frequency and quantity	4
Flatulence	3
Hyperpigmentation of the skin	3
Muscle problems including pain	3
Brain fog	2
Dizziness	2
Frequent visits to the toilet	2
Nail problems	2
Fear of recurrence	2
Sensitivity to cold temperatures	2
Worry about the impact on others	2
Bad taste in the mouth	1
Feeling hungry	1
General skin problems	1
Hair color change (graying)	1
Hiccups	1
Edema	1
Sensitivity to smells	1
Sight problems	1
Sweating after meals	1
Worry about eating in front of others	1
Worry about surgery	1
Worry about the stigma of having cancer	1
Worry about death	1
Wound healing difficulties	1

### Phase 1b patient and HCP interviews

#### Sample

Forty-three patients were involved in a second phase (1b) of interviews and included patients from the UK (*n* = 9), Japan (*n* = 8), Korea (*n* = 5), China (*n* = 5), Mongolia (*n* = 5), Turkey (*n* = 5), India (*n* = 3), and Spain (*n* = 3). The sample matches the Phase 1a sample in terms of representativeness (online resource 4). Eighteen HCPs also participated and included 9 males, and 6 females (aged between 23 and 57) from different disciplines with over half (56%) having at least 5 years’ experience in treating gastric cancer and half from countries within East Asia.

#### Ratings of existing QLQ-STO22 questions and further feedback.

Relevance ratings for existing QLQ-STO22 questions ranged from 37% (hair loss) to 77% (worries about future health) for patients and 100% (problems eating food (solid or liquidized)) to 50% (dry mouth) for HCPs (Table 2). All questions were rated on average as at least “a little bit” important by both patients and HCPs with the lowest mean scores for dry mouth (1.70 and 1.83) and the highest for problems with belching (2.74 for patients) and problems with eating solid foods (3.61 for HCPs). Worries about future health and problems eating solid foods were rated as the highest priority questions by patients and HCPs respectively (39.5 and 66.7%). No more than three patients and four HCPs recommended any one item for removal from the original questionnaire. Problems eating liquidized or soft food, eating in front of others, and being upset by hair loss were the most frequently nominated questions for exclusion by patients, while dry mouth was nominated by HCPs.

**Table 2:** Participant (*n* = 43 patients, *n* = 18 HCPs) ratings for the QLQ-STO22 questions

QLQ-STO22 Question	Relevance		Importance		Priority		Exclude	
	Number (%) Patients	Number (%) HCPs	Mean (SD) Patients	Mean (SD) HCPs	Number (%) Patients	Number (%) HCPs	Number (%) Patients	Number (%) HCPs
Have you had problems eating solid foods?	29 (67%)	18 (100%)	2.16 (1.15)	3.61 (0.78)	14 (33%)	12 (67%)	1 (2%)	0
Have you had problems eating liquidized or soft foods?	22 (51%)	18 (100%)	2.23 (1.17)	3.28 (0.89)	11 (26%)	7 (39%)	3 (7%)	0
Have you had problems drinking liquids?	23 (54%)	17 (94%)	2.30 (1.30)	2.83 (0.99)	11 (26%)	6 (33%)	1 (2%)	0
Have you had discomfort when eating?	25 (58%)	16 (89%)	2.35 (1.23)	3.35 (0.86)	9 (21%)	9 (50%)	2 (5%)	0
Have you had pain in your stomach area?	20 (47%)	17 (94%)	2.56 (1.30)	3.33 (0.91)	14 (33%)	10 (56%)	0	1 (6%)
Have you had discomfort in your stomach area?	24 (56%)	17 (94%)	2.67 (1.26)	3.11 (0.83)	8 (19%)	7 (39%)	0	0
Did you have a bloated feeling in your abdomen?	28 (65%)	17 (94%)	2.63 (1.27)	3.11 (0.90)	11 (26%)	9 (50%)	0	0
Have you had trouble with acid or bile coming into your mouth?	26 (61%)	14 (78%)	2.19 (1.16)	2.88 (1.02)	11 (26%)	7 (39%)	0	1 (6%)
Have you had acid indigestion or heartburn?	23 (54%)	15 (83%)	2.56 (1.14)	3.00 (1.06)	8 (19%)	7 (39%)	0	0
Have you had trouble with belching?	26 (61%)	15 (83%)	2.74 (1.04)	2.50 (0.86)	6 (14%)	2 (11%)	2 (5%)	0
Have you felt full up to quickly after beginning to eat?	31 (72%)	17 (94%)	2.47 (1.08)	3.44 (0.78)	12 (28%)	11 (61%)	0	0
Have you had trouble enjoying your meals?	31 (72%)	16 (89%)	2.33 (1.19)	3.33 (0.97)	11 (26%)	8 (44%)	1 (2%)	0
Has it taken you a long time to complete your meals?	32 (74%)	14 (78%)	2.48 (1.21)	2.94 (1.16)	6 (14%)	6 (33%)	1 (2%)	2 (11%)
Have you had a dry mouth?	29 (67%)	9 (50%)	1.70 (0.96)	1.83 (0.92)	8 (19%)	0	1 (2%)	4 (22%)
Did food and drink taste different to usual?	27 (63%)	13 (72%)	2.58 (1.22)	2.56 (1.04)	11 (26%)	4 (22%)	0	1 (6%)
Have you had trouble with eating in front of other people?	13 (30%)	11 (61%)	2.58 (1.22)	2.33 (0.97)	2 (5%)	2 (11%)	3 (7%)	1 (6%)
Have you been thinking about your illness?	27 (63%)	15 (83%)	2.53 (1.26)	2.89 (1.08)	14 (33%)	6 (33%)	1 (2%)	3 (17%)
Have you worried about your weight being too low?	28 (65%)	17 (94%)	2.88 (1.22)	2.94 (1.00)	11 (26%)	11 (61%)	1 (2%)	1 (6%)
Have you felt physically less attractive as a result of your disease or treatment?	26 (61%)	12 (67%)	2.33 (1.25)	2.56 (0.92)	7 (16%)	4 (22%)	2 (5%)	1 (6%)
Have you worried about your health in future?	33 (77%)	16 (89%)	1.91 (1.23)	3.06 (1.11)	17 (40%)	6 (33%)	0	0
Were you upset by hair loss?	16 (73%)*	11 (61%)	2.40 (1.22)	2.12 (0.99)	1 (2%)	0	3 (7%)	2 (11%)

^*^Percentage calculated from those who had lost hair (*n* = 22)

In terms of feedback on the questions flagged as potentially confusing during the first round of interviews, most patients did not report any problems with comprehension, and there was an overall preference for the original wording over the proposed alternatives (for bloating and belching) written following earlier feedback (Table 3). In addition, only three patients reported that the question about physical appearance was potentially upsetting; one patient from South Korea mentioned: “It is not upsetting, but it is important to me”, and another patient from the UK explained: “There is no better way of asking, but it needs to be asked”. There was a tendency for more HCPs compared with patients to anticipate problems with comprehension and to rate the physical attractiveness question as potentially upsetting. In terms of any missing questions from the list, one HCP and one patient mentioned that there needs to be more consideration of chemotherapy-related effects such as neuropathy.

**Table 3:** Patient and HCP feedback on potentially confusing and upsetting QLQ-STO22 questions

	Patients (*N* = 43)	HCPs (*N* = 18)
*Confusing* Did you have a bloated feeling in your abdomen? *Prefer alternative* Is retaining gas a problem for you?	9 (21%) 7 (16%)	4 (22%) 4 (22%)
*Confusing* Have you had trouble with belching? *Prefer alternative* Is belching a problem for you?	7 (16%) 5 (12%)	7 (39%) 5 (28%)
*Confusing* Have you had problems eating solid food?	9 (21%)	3 (17%)
*Confusing* Have you had trouble with eating in front of people?	4 (9%)	6 (33%)
*Upsetting* Have you felt physically less attractive as a result of your disease or treatment?	3 (7%)	3 (17%)

#### Ratings of new issues

Of the 58 new issues (from the 26 broad areas of HRQoL concern presented in Table 1), those relating to eating (feeling full, eating small amounts, and eating many times per day) were rated the most relevant (65% to 74% of patients; Table 4). This finding was also echoed in HCPs ratings: 17/18 identified distress when eating, inability to eat a meal due to fullness and the feeling of food not going down easily as relevant to the patients they treat. In addition, 17 HCPs reported flatulence and increased frequency of visiting the toilet as relevant. Eating smaller amounts and inability to eat a meal due to fullness were also rated as priority issues to include by 21% of patients each and 44% and 50% of HCPs, respectively, while flatulence was a priority question for 14% of patients and 44% of HCPs.

**Table 4:** Participant (*n *= 43 patients, *n* = 18 HCPs) ratings for the new issues

Issue	Relevance		Importance		Priority		Exclude	
	Number (%) Patients	Number (%) HCPs	Mean (SD) Patients	Mean (SD) HCPs	Number (%) Patients	Number (%) HCPs	Number (%) Patients	Number (%) HCPs
Bad taste in the mouth	20 (47%)	14 (78%)	1.52 (0.86)	2.61 (1.09)	2 (5%)	1 (6%)	0	0
Brain fog	12 (28%)	11 (61%)	1.72 (1.08)	2.18 (1.07)	2 (5%)	3 (17%)	3 (7%)	0
Bone pain (bottom of the spine)	6 (14%)	6 (33%)	1.45 (0.94)	1.50 (0.9)	1 (2%)	0	0	2 (11%)
Burning sensation of the feet	13 (30%)	13 (72%)	1.67 (0.98)	2.33 (1.03)	4 (9%)	2 (11%)	1 (2%)	0
Choking when swallowing	13 (30%)	11 (61%)	1.57 (0.86)	2.22 (1.11)	3 (7%)	3 (17%)	0	0
Concerns about treatment efficacy	20 (47%)	15 (83%)	2.05 (1.17)	2.78 (0.93)	1 (2%)	3 (7%)	1 (2%)	0
Cough	9 (21%)	11 (61%)	1.40 (0.79)	1.72 (0.75)	2 (5%)	1 (6%)	3 (7%)	0
Crusted wounds	9 (21%)	12 (67%)	1.52 (0.89)	1.89 (0.76)	3 (7%)	0	2 (5%)	0
Difficulty hearing	8 (19%)	3 (17%)	1.55 (0.97)	1.33 (0.69)	5 (12%)	0	3 (7%)	0
Difficulty remembering to take medication	11 (26%)	11 (61%)	1.65 (1.00)	2.67 (0.91)	0	0	1 (2%)	0
Difficulty speaking	6 (14%)	5 (28%)	1.52 (0.86)	1.44 (0.70)	1 (2%)	0	2 (5%)	1 (6%)
Difficulty swallowing	16 (37%)	16 (89%)	1.44 (0.81)	1.94 (0.97)	4 (9%)	3 (17%)	0	0
Difficulty swallowing saliva	16 (37%)	15 (83%)	1.71 (0.97)	2.28 (0.83)	3 (7%)	0	0	0
Difficulty taking medication	12 (28%)	15 (83%)	1.58 (0.91)	2.67 (0.83)	0	1 (6%)	1 (2%)	0
Distress when eating	12 (28%)	17 (94%)	1.76 (1.05)	2.72 (0.83)	1 (2%)	0	1 (2%)	0
Dizziness or vertigo	17 (40%)	11 (61%)	1.66 (1.06)	3.00 (0.97)	6 (14%)	0	2 (5%)	0
Eating smaller quantities	32 (74%)	16 (89%)	2.29 (1.11)	2.63 (1.15)	9 (21%)	8 (44%)	0	0
Fainting	10 (23%)	2 (11%)	2.60 (1.06)	3.17 (1.04)	0	1 (6%)	3 (7%)	0
Fear of death	20 (47%)	16 (89%)	1.62 (0.96)	2.40 (1.52)	2 (5%)	6 (33%)	1 (2%)	0
Fear of surgery	16 (37%)	14 (78%)	1.74 (0.96)	2.67 (1.03)	1 (2%)	2 (11%)	1 (2%)	1 (6%)
Feeling cold	18 (42%)	13 (72%)	1.88 (1.07)	2.33 (0.97)	3 (7%)	0	2 (5%)	0
Fever	7 (16%)	12 (67%)	1.63 (0.98)	2.06 (1.06)	0	3 (17%)	2 (5%)	0
Flatulence	24 (56%)	17 (94%)	2.49 (1.18)	3.06 (0.87)	6 (14%)	8 (44%)	0	0
Feeling that food does not go down easily	21 (49%)	17 (94%)	2.19 (1.13)	3.00 (0.84)	3 (7%)	5 (28%)	1 (2%)	0
Feeling of food getting stuck in the throat	12 (28%)	14 (78%)	1.81 (1.17)	2.67 (1.03)	4 (9%)	4 (22%)	1 (2%)	0
Hair color graying	13 (30%)	5 (28%)	1.70 (1.06)	1.33 (0.49)	0	0	2 (5%)	1 (6%)
Headache	4 (9%)	9 (50%)	1.37 (0.85)	1.78 (0.88)	0	0	3 (7%)	0
Hiccups	13 (30%)	15 (83%)	1.70 (1.01)	2.33 (0.97)	6 (14%)	4 (22%)	2 (5%)	0
Hot flushes	7 (16%)	8 (44%)	1.52 (0.89)	1.59 (0.71)	0	0	2 (5%)	0
Hunger outside of mealtimes	13 (30%)	12 (68%)	1.69 (1.02)	2.35 (1.17)	4 (9%)	2 (11%)	0	0
Hunger pain	17 (40%)	12 (68%)	1.90 (1.05)	2.33 (1.14)	5 (12%)	0	0	1 (6%)
Inability to eat a meal due to fullness	28 (65%)	17 (94%)	2.47 (1.08)	3.33 (0.97)	9 (21%)	9 (50%)	0	0
Increased appetite	15 (35%)	7 (39%)	1.67 (0.99)	1.71 (0.77)	1 (2%)	0	1 (2%)	2 (11%)
Increased frequency of eating	29 (67%)	15 (83%)	1.88 (0.98)	2.22 (1.17)	6 (14%)	4 (22%)	0	0
Increased frequency of needing to use the toilet	22 (51%)	17 (94%)	2.12 (1.19)	2.94 (0.94)	4 (9%)	5 (28%)	0	0
Increased heart rate	6 (14%)	12 (67%)	1.43 (0.83)	2.12 (0.93)	1 (2%)	0	2 (5%)	0
Intolerance of certain foods	20 (47%)	16 (89%)	2.14 (1.18)	2.78 (0.88)	3 (7%)	1 (6%)	0	0
Intolerance of certain smells	17 (40%)	14 (78%)	1.84 (1.11)	2.44 (0.98)	1 (2%)	1 (6%)	0	0
Itchy skin	9 (21%)	7 (39%)	1.48 (0.89)	1.44 (0.62)	2 (5%)	0	1 (2%)	0
Inflammation of the lips and mouth	11 (26%)	13 (72%)	1.48 (0.89)	2.28 (0.89)	3 (7%)	2 (11%)	0	0
Lack of enjoyment of certain foods	20 (47%)	13 (72%)	2.14 (1.18)	2.65 (1.11)	5 (12%)	3 (17%)	0	0
Light headedness	15 (35%)	11 (61%)	1.78 (0.94)	2.39 (1.14)	5 (12%)	1 (6%)	3 (7%)	0
Loss of independence	20 (47%)	15 (83%)	1.98 (1.12)	2.44 (0.98)	4 (9%)	2 (11%)	1 (2%)	1 (6%)
Muscle pain	10 (23%)	14 (78%)	1.72 (0.98)	2.28 (0.89)	3 (7%)	1 (6%)	0	0
Nail loss or nail disease	7 (16%)	14 (78%)	1.40 (0.91)	2.11 (0.83)	3 (7%)	1 (6%)	0	0
Noises or rumbles in the stomach	22 (51%)	16 (89%)	2.05 (1.05)	2.56 (0.92)	5 (12%)	2 (11%)	0	0
Numbness	11 (26%)	12 (67%)	1.83 (1.17)	2.39 (1.14)	5 (12%)	3 (7%)	1 (2%)	0
Red skin reaction	7 (16%)	10 (56%)	1.56 (1.05)	1.83 (0.86)	5 (12%)	1 (6%)	0	0
Retching as if to vomit but with no production	19 (44%)	15 (83%)	2.16 (1.25)	2.89 (1.13)	3 (7%)	6 (33%)	0	0
Skin color changes	8 (19%)	10 (56%)	1.44 (0.93)	1.94 (1.00)	3 (7%)	1 (6%)	1 (2%)	2 (11%)
Shaking or tremors	13 (30%)	2 (11%)	1.69 (0.95)	1.60 (0.89)	4 (9%)	0	3 (7%)	0
Speech difficulties	6 (14%)	5 (28%)	1.36 (0.73)	1.59 (0.87)	3 (7%)	0	0	0
Stomach cramps	11 (26%)	16 (89%)	1.62 (1.03)	2.64 (0.63)	2 (5%)	1 (6%)	0	0
Sweating after a meal	11 (26%)	15 (83%)	1.55 (0.86)	2.33 (0.84)	1 (2%)	1 (6%)	1 (2%)	0
Swelling of the lower legs, feet or ankles	8 (19%)	15 (83%)	1.48 (0.80)	2.50 (0.92)	3 (7%)	3 (7%)	1 (2%)	0
Urgency when needing the toilet	19 (44%)	16 (89%)	2.00 (1.13)	2.56 (0.92)	3 (7%)	2 (11%)	1 (2%)	0
Visual difficulties	9 (21%)	5 (28%)	1.47 (0.86)	1.44 (0.70)	2 (5%)	1 (6%)	2 (5%)	0
Weakness in lower limbs	21 (49%)	17 (94%)	2.19 (1.14)	2.78 (0.81)	5 (12%)	1 (6%)	0	0
Wound healing problems	13 (30%)	15 (83%)	1.67 (1.03)	2.28 (0.83)	3 (7%)	0	2 (5%)	0

### Cross-cultural comparisons

Participants from all countries proposed suggestions for improvements to the QLQ-STO22 questions and potential new issues, and, thus no clear patterns in terms of feedback content according to cultural background were captured. In the second set of interviews, participants from both East Asian and non-East Asian countries shared their opinions on the body image question. Of the three patient participants who mentioned that it was potentially upsetting, one was from non-East Asia. Patients from East Asia and non-East Asia were comparable in terms of their ratings of QLQ-STO22 questions for relevance and importance (Table 5). The mean importance ratings of 13 of the 22 questions were higher for patients from East Asia. Of particular importance (and relevance) to patients from East Asia include questions about feeling less attractive, illness cognitions, and future health concerns.

**Table 5:** Comparison of patient relevance and importance ratings according to geographical location (East Asia *n* = 23, outside East Asia *n* = 20)

	**Patient group**	**Relevance** **Number (percentage)**	**Importance** **Mean (standard deviation)**
STO22Q31. Have you had problems eating solid foods?	East Asia	14 (61%)	2.52 (1.24)
	Non-East Asia	15 (75%)	2.70 (1.17)
STO22Q32. Have you had problems eating liquidized or soft foods?	East Asia	10 (44%)	2.35 (1.23)
	Non-East Asia	12 (60%)	1.95 (1.05)
STO22Q33. Have you had problems drinking liquids?	East Asia	10 (44%)	2.43 (1.24)
	Non-East Asia	13 (65%)	2.00 (1.08)
STO22Q34. Have you had discomfort when eating?	East Asia	12 (52%)	2.43 (1.24)
	Non-East Asia	13 (65%)	2.35 (1.23)
STO22Q35. Have you had pain in your stomach area?	East Asia	10 (44%)	2.43 (1.24)
	Non-East Asia	10 (50%)	2.15 (1.39)
STO22Q36. Have you had discomfort in your stomach area?	East Asia	12 (52%)	2.43 (1.20)
	Non-East Asia	12 (60%)	2.25 (1.29)
STO22Q37. Did you have a bloated feeling in your abdomen?	East Asia	13 (57%)	2.48 (1.31)
	Non-East Asia	15 (75%)	2.65 (1.31)
STO22Q38. Have you had trouble with acid or bile coming into your mouth?	East Asia	11 (48%)	2.43 (1.16)
	Non-East Asia	15 (75%)	2.95 (1.35)
STO22Q39. Have you had acid indigestion or heartburn?	East Asia	8 (35%)	2.43 (1.27)
	Non-East Asia	15 (75%)	2.85 (1.27)
STO22Q40. Have you had trouble with belching?	East Asia	12 (52%)	2.09 (1.20)
	Non-East Asia	14 (70%)	2.30 (1.13)
STO22Q41. Have you felt full up to quickly after beginning to eat?	East Asia	14 (61%)	2.61 (1.20)
	Non-East Asia	17 (85%)	2.50 (1.10)
STO22Q42. Have you had trouble enjoying your meals?	East Asia	14 (61%)	2.61 (1.12)
	Non-East Asia	17 (85%)	2.90 (1.02)
STO22Q43. Has it taken you a long time to complete your meals?	East Asia	15 (63%)	2.52 (1.12)
	Non-East Asia	17 (85%)	2.40 (1.05)
STO22Q44. Have you had a dry mouth?	East Asia	14 (61%)	2.13 (1.06)
	Non-East Asia	15 (75%)	2.55 (1.32)
STO22Q45. Did food and drink taste different to usual?	East Asia	12 (52%)	2.30 (1.18)
	Non-East Asia	15 (75%)	2.68 (1.25)
STO22Q46. Have you had trouble with eating in front of other people?	East Asia	9 (39%)	1.96 (0.88)
	Non-East Asia	4 (20%)	1.40 (0.99)
STO22Q47. Have you been thinking about your illness?	East Asia	19 (83%)	2.96 (1.07)
	Non-East Asia	8 (40%)	2.15 (1.27)
STO22Q48. Have you worried about your weight being too low?	East Asia	15 (65%)	2.70 (1.22)
	Non-East Asia	13 (65%)	2.45 (1.23)
STO22Q49. Have you felt physically less attractive as a result of your disease or treatment?	East Asia	16 (70%)	3.09 (1.12)
	Non-East Asia	10 (50%)	1.90 (1.12)
STO22Q50. Have you worried about your health in future?	East Asia	21 (91%)	3.35 (0.88)
	Non-East Asia	12 (60%)	2.35 (1.35)
STO22Q52. Answer this question only if you have lost hair: were you upset by the loss of your hair?	East Asia	9 (39%)	2.13 (1.29)
	Non-East Asia	7 (35%)	1.65 (1.14)

### Expert panel review

None of the QLQ-STO22 questions were identified as candidates for removal based on importance and relevance thresholds for exclusion. In terms of linguistic changes, it was recommended to replace “problems” with “trouble” and “Did you” with “Have you” to allow for consistency in how the questions are posed. No culturally specific changes were identified.

The following three new issues met the criteria for inclusion: Eating small amounts, flatulence and not being able to eat a meal because of fullness or becoming full quickly. However, the issue of fullness was regarded as overlapping in content with the current QLQ-STO22 question on feeling full up too quickly after beginning to eat. The issue of neuropathy, identified in the first set of interviews as an important omission, did not emerge as particularly relevant or important in the second set of interviews when explored as numbness, tingling, or weakness. However, clinical experts recommended that neuropathy should be included, given its prominence within the symptom side-effect profile of newer chemotherapy regimens (e.g., those using oxaliplatin) and that it is commonly presented as a debilitating issue for patients with metastatic disease.

## Discussion

Our study invited 104 patients from diverse cultural backgrounds, including 48 from countries within East Asia, to share their experiences of GC and review a well-established and validated HRQoL measure for this patient group, the QLQ-STO22. Eighteen HCPs also shared their feedback on the QLQ-STO22. The results from this study provide support for the cross-cultural applicability and acceptability of the QLQ-STO22 as well as confirming that the QLQ-STO22 remains relevant and important. There was a reluctance among patients and HCPs to nominate any of the existing questions for removal, and even when the question about physical attractiveness was explored in follow-up interviews as a question of potential concern, especially among patients from East Asia, this was not substantiated and indeed, was highlighted as a question of particular importance and relevance.

The study also identified additional questions to be addressed by the QLQ-ST022. An exhaustive list of issues was presented to participants, many of which were captured during the earlier development [16] but excluded based on the robust EORTC QLG decision rules applied. Based on rating scores and feedback, three issues (two relating to eating and one describing flatulence) were considered for inclusion although after careful consideration, one of the eating issues was regarded as overlapping in content with an existing QLQ-STO22 question. A further issue relating to neuropathy was highlighted for inclusion based on patient and HCP feedback and subsequent clinical expert review. The neuropathy issues did not meet the inclusion criteria based on importance, relevance, and priority nominations, but this might be explained by the splitting of the neuropathy issue into three separate constructs: tingling sensation, burning, and weakness and that patients included in the sample might be at a lower risk of neuropathy given their limited exposure to chemotherapies. Unfortunately, this study did not collect information relating to the number of chemotherapy cycles, which would have afforded valuable insight into neuropathy.

The three issues recommended for addition to the existing QLQ-STO22 were formulated into the following five questions: Have you been restricted in the amounts of food you could eat as a result of your disease or treatment? Have you had problems with gas (flatulence)? Have you had numbness in your fingers or toes? Have you had shooting or burning pain in your toes or feet? Have you had difficulty climbing stairs or getting out of a chair because of weakness in your legs? We were mindful that patients emphasized that the QLQ-STO22 is already lengthy and therefore avoidance of additional patient burden needs to be respected.

### Strengths and limitations

One of the primary objectives of this study was to assess whether the QLQ-STO22 is suitable for international use including in countries within East Asia where it is arguably most needed. The study involved patients and specialists in GC from 10 countries, four from within East Asia, three in Europe, and three outside Europe, which contrasts with eight countries (six within Europe and two Westernized non-European countries) involved in the development and validation studies of the QLQ-STO22 [16, 17]. Thus, this study allows for more in-depth scrutiny of the HRQoL issues affecting people with GC across diverse cultural backgrounds, considering their different value and belief systems as well as treatment practices. The methodology of the study was embedded within the EORTC QLG’s robust and rigorous framework for questionnaire development [15], which emphasizes the patient’s voice. Although the opinion of HCPs and clinical experts was considered, our recommendations have been largely shaped by what the patients tell us is important to ask them. A certain degree of discordance between HCP and patient ratings, e.g., with a tendency for more HCPs than patients themselves to rate existing QLQ-STO22 questions as potentially upsetting, further highlights the value of prioritizing the patient’s perspective, which is a key strength of this study.

Although this study achieved its target recruitment with a good representation of participants from East Asia and non-East Asia, cultural sub-group comparisons were confined to descriptive analyses as the sample sizes did not afford enough power to conduct robust statistical tests of significance. In addition, data on chemotherapy regimens were limited. Hence, we are not able to fully explore chemotherapy-related issues such as neuropathy.

### Future plans

The updated version of the questionnaire will need testing with a larger sample of patients, including those from East Asia, followed by an international validation study to ensure robust psychometric testing of items and subscales. It is acknowledged that modifications to a well-established questionnaire such as the QLQ-STO22 could introduce confusion as to the selection of the optimal measure to use and might also compromise the ability to draw comparisons across studies using different versions of the measure. We would like to confirm that the QLQ-STO22 can still be used in trials.

## Conclusions

The QLQ-STO22 is one of the most widely used measures to assess HRQoL in people with GC. This study represents an extension of its initial development and validation work, offering further support for the cross-cultural relevance and acceptability of the QLQ-STO22. Recommendations to improve the sensitivity of the measure have been proposed. The proposed updated questionnaire will undergo further psychometric testing to ensure that the patient experience of GC is measured in the most precise way.

### Supplementary Information

Below is the link to the electronic supplementary material.

Supplementary file1 (DOCX 81 KB)
