# Supplementary material for: Revisiting the use of the EORTC QLQ-STO22 to assess health-related quality of life of patients with gastric cancer: incorporating updated treatment options and cross-cultural perspectives
Source: Gastric Cancer. 2024 Apr 26;27(4):722–34. doi: 10.1007/s10120-024-01492-8 (PMC11193686; doi:10.1007/s10120-024-01492-8)
Supplement: Supplementary file 1 — Supplementary file1 (DOCX 81 KB) [file 10120_2024_1492_MOESM1_ESM.docx]

**Online resources**

## Online resource 1

## Phase 1a patient interview schedule

[
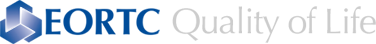
](http://groups.eortc.be/qo)

**Health-related Quality of Life in Adults with Stomach Cancer**

**Phase 1a Interview Guide for Updating the EORTC QLQ-STO22 stomach cancer module.**

### Opening

1. Please can you tell me about how cancer and its treatment has affected your life?

PROMPTS [Some patients have mentioned ……., what about the impact on being able to go out and socialise? How has it affected you emotionally? Treatment burden?]

1. I am going to show you two questionnaires [ show the patient the EORTC QLQ-30 first and then the STO22]. Each has a list of questions that may or may not be relevant to you. Is there anything on these questionnaires which you think are relevant to you and which we have not talked about? [*Note for researcher or clinician: Encourage patients to “think aloud” and expand on their accounts*].

### Review of EORTC QLQ-STO22

*Ask the following questions in relation to the EORTC QLQ-STO22*

1. Are there any questions on the STO22 which are not relevant?
2. Are any questions of the questions confusing or difficult to answer?
3. Can you explain what was confusing or difficult about the questions?
4. Do you think any of the questions are upsetting?
5. Can you explain what is upsetting about these questions?
6. Are there any issues missing from the questionnaire?

### Summary and closure

1. *Summarise key points of the discussion*: You have told me the different ways in which cancer and its treated has affected you….. Of the issues you have mentioned, what do you think have been the most important or troubling to you?
2. Is there anything else that we have not covered that you would like to share with me regarding your experience of stomach cancer and its treatment?
3. Thank you for your time and for sharing your experiences. If you would like to find out more about the study and the results, you are more than welcome to look on the EORTC Quality of Life Group *(remind participant of address on Participant Information Sheet).*

## Online resource 2: Phase 1b interview schedule for Patients and Health Care Professionals

[
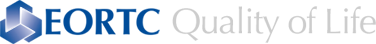
](http://groups.eortc.be/qo)

**Health-related Quality of Life in Adults with Gastric Cancer**

**Phase 1b Interview Guide for Updating the EORTC QLQ-STO22 gastric cancer module**

### Opening

1. Please can you tell me about your experience of having cancer and receiving treatment / the patients you treat / look after who have gastric cancer. How does cancer and its treatment affect you life / their lives?
2. PROMPTS [Some patients we have spoken to have mentioned ……., what about the impact on being able to go out and socialise? The emotional impact? Treatment burden?]

### Introduction to EORTC QLQ-STO22

1. I am going to show you two questionnaires *[ show the patient the EORTC QLQ-30 first and then the STO22*]. Each has a list of questions that may or may not be relevant to the patients you see. Are there any questions on these questionnaires that you recognise as important for this patient group?

[*Note for researcher or clinician: Encourage participants to “think aloud” and expand on their accounts*].

### Rating the EORTC QLQ-STO22 for relevance

1. Now I would like you to look at the questions on this questionnaire which was developed specifically for people with gastric cancer (*Show the EORTC QLQ-STO22 and ask the participant to rate each question*). I would like you to rate these questions according to ‘Relevance’ and ‘Importance’:

**‘Relevance’** means whether the issue is something you recognise as happening to you (or patients you treat) at some point since your diagnosis and as a result of stomach cancer / treatment.

**‘Importance’** relates to the degree to which the issue has troubled you or been bothersome to you (or patients you treat).

Could you please indicate for each question separately the extent to which you find it relevant (either now or in the past) for you in terms of:

NO YES

And the extent to which you find it important in terms of:

1. Not at all important
2. A little important
3. Quite a bit important
4. Very much important

**Please think about each question in terms of stomach cancer / its treatment**

|  | **RELEVANT?** | | **IMPORTANT?** | | | | | **10 Most Important (√)** | **Do not include (√)** |
| --- | --- | --- | --- | --- | --- | --- | --- | --- | --- |
|  | **NO** | **YES** | **Not at**  **all** | | **A little** | **Quite a**  **bit** | **Very**  **much** |  |  |
| **Symptoms from the STO22** |  |  |  |  | |  |  |  |  |
| Q31. Have you had problems eating solid foods? |  |  | 1 | 2 | | 3 | 4 |  |  |
| Q32. Have you had problems eating liquidised or soft foods? |  |  | 1 | 2 | | 3 | 4 |  |  |
| Q33. Have you had problems drinking liquids? |  |  | 1 | 2 | | 3 | 4 |  |  |
| Q34. Have you had discomfort when eating? |  |  | 1 | 2 | | 3 | 4 |  |  |
| Q35. Have you had pain in your stomach area? |  |  | 1 | 2 | | 3 | 4 |  |  |
| Q36. Have you had discomfort in your stomach area? |  |  | 1 | 2 | | 3 | 4 |  |  |
| Q37. Did you have a bloated feeling in your abdomen? |  |  | 1 | 2 | | 3 | 4 |  |  |
| Q38. Have you had trouble with acid or bile coming into your mouth? |  |  | 1 | 2 | | 3 | 4 |  |  |
| Q39. Have you had acid indigestion or heartburn? |  |  | 1 | 2 | | 3 | 4 |  |  |
| Q40. Have you had trouble with belching? |  |  | 1 | 2 | | 3 | 4 |  |  |
| Q41. Have you felt full up to quickly after beginning to eat? |  |  | 1 | 2 | | 3 | 4 |  |  |
| Q42. Have you had trouble enjoying your meals? |  |  | 1 | 2 | | 3 | 4 |  |  |
| Q43. Has it taken you a long time to complete your meals? |  |  | 1 | 2 | | 3 | 4 |  |  |
| Q44. Have you had a dry mouth? |  |  | 1 | 2 | | 3 | 4 |  |  |
| Q45. Did food and drink taste different to usual? |  |  | 1 | 2 | | 3 | 4 |  |  |
| Q46. Have you had trouble with eating in front of other people? |  |  | 1 | 2 | | 3 | 4 |  |  |
| Q47. Have you been thinking about your illness? |  |  | 1 | 2 | | 3 | 4 |  |  |
| Q48. Have you worried about your weight being too low? |  |  | 1 | 2 | | 3 | 4 |  |  |
| Q49. Have you felt physically less attractive as a result of your disease or treatment? |  |  | 1 | 2 | | 3 | 4 |  |  |
| Q50. Have you worried about your health in the future? |  |  | 1 | 2 | | 3 | 4 |  |  |
| Q51. Have you lost any hair? |  |  | 1 | 2 | | 3 | 4 |  |  |
| Q52. Answer this question only if you have lost hair:  If so, were you upset by the loss of your hair? |  |  | 1 | 2 | | 3 | 4 |  |  |

### Rating the list of concerns from Phase 1a interviews for relevance

1. We have also carried out interviews with people with gastric cancer and from these interviews we have drawn up a list of concerns (*present list*). We would like you to think about whether each of these concerns are relevant to the patients you see.

Could you please indicate for each concern separately the extent to which you find it relevant (either now or in the past) for you in terms of:

NO YES

And the extent to which you find it important in terms of:

1. Not at all important
2. A little important
3. Quite a bit important
4. Very much important

|  | **RELEVANT?** | | **IMPORTANT?** | | | | **Comments** | **10 Most Important (√)** | **Do not include (√)** |
| --- | --- | --- | --- | --- | --- | --- | --- | --- | --- |
|  | **NO** | **YES** | **Not at**  **all** | **A little** | **Quite a**  **bit** | **Very**  **much** |  |  |  |
| **New issues list** |  |  |  |  |  |  |  |  |  |
| Bad taste in the mouth |  |  | 1 | 2 | 3 | 4 |  |  |  |
| Brain fog |  |  | 1 | 2 | 3 | 4 |  |  |  |
| Bone pain (bottom of the spine) |  |  | 1 | 2 | 3 | 4 |  |  |  |
| Burning sensation of the feet |  |  | 1 | 2 | 3 | 4 |  |  |  |
| Choking when swallowing |  |  | 1 | 2 | 3 | 4 |  |  |  |
| Concerns about treatment efficacy |  |  | 1 | 2 | 3 | 4 |  |  |  |
| Cough |  |  | 1 | 2 | 3 | 4 |  |  |  |
| Crusted wounds |  |  | 1 | 2 | 3 | 4 |  |  |  |
| Difficulty hearing |  |  | 1 | 2 | 3 | 4 |  |  |  |
| Difficulty remembering |  |  | 1 | 2 | 3 | 4 |  |  |  |
| Difficulty speaking |  |  | 1 | 2 | 3 | 4 |  |  |  |
| Difficulty swallowing |  |  | 1 | 2 | 3 | 4 |  |  |  |
| Difficulty swallowing saliva |  |  | 1 | 2 | 3 | 4 |  |  |  |
| Difficulty taking medication |  |  | 1 | 2 | 3 | 4 |  |  |  |
| Distress when eating |  |  | 1 | 2 | 3 | 4 |  |  |  |
| Dizziness or vertigo |  |  | 1 | 2 | 3 | 4 |  |  |  |
| Eating smaller quantities |  |  | 1 | 2 | 3 | 4 |  |  |  |
| Fainting |  |  | 1 | 2 | 3 | 4 |  |  |  |
| Fear of death |  |  | 1 | 2 | 3 | 4 |  |  |  |
| Fear of surgery |  |  | 1 | 2 | 3 | 4 |  |  |  |
| Feeling cold |  |  | 1 | 2 | 3 | 4 |  |  |  |
| Fever |  |  | 1 | 2 | 3 | 4 |  |  |  |
| Flatulence |  |  | 1 | 2 | 3 | 4 |  |  |  |
| Feeling that food does not go down easily |  |  | 1 | 2 | 3 | 4 |  |  |  |
| Feeling of food getting stuck in the throat |  |  | 1 | 2 | 3 | 4 |  |  |  |
| Hair colour greying |  |  | 1 | 2 | 3 | 4 |  |  |  |
| Headache |  |  | 1 | 2 | 3 | 4 |  |  |  |
| Hiccups |  |  | 1 | 2 | 3 | 4 |  |  |  |
| Hot flushes |  |  | 1 | 2 | 3 | 4 |  |  |  |
| Hunger outside of mealtimes |  |  | 1 | 2 | 3 | 4 |  |  |  |
| Hunger pain |  |  | 1 | 2 | 3 | 4 |  |  |  |
| Inability to eat a meal due to fullness |  |  | 1 | 2 | 3 | 4 |  |  |  |
| Increased appetite |  |  | 1 | 2 | 3 | 4 |  |  |  |
| Increased frequency of eating |  |  | 1 | 2 | 3 | 4 |  |  |  |
| Increased frequency of needing to use the toilet |  |  | 1 | 2 | 3 | 4 |  |  |  |
| Increased heart rate |  |  | 1 | 2 | 3 | 4 |  |  |  |
| Intolerance of certain foods |  |  | 1 | 2 | 3 | 4 |  |  |  |
| Intolerance of certain smells |  |  | 1 | 2 | 3 | 4 |  |  |  |
| Itchy skin |  |  | 1 | 2 | 3 | 4 |  |  |  |
| Inflammation of the lips and mouth |  |  | 1 | 2 | 3 | 4 |  |  |  |
| Lack of enjoyment of certain foods |  |  | 1 | 2 | 3 | 4 |  |  |  |
| Light headedness |  |  | 1 | 2 | 3 | 4 |  |  |  |
| Loss of independence |  |  | 1 | 2 | 3 | 4 |  |  |  |
| Muscle pain |  |  | 1 | 2 | 3 | 4 |  |  |  |
| Nail loss or nail disease |  |  | 1 | 2 | 3 | 4 |  |  |  |
| Noises or rumbles in the stomach |  |  | 1 | 2 | 3 | 4 |  |  |  |
| Numbness |  |  | 1 | 2 | 3 | 4 |  |  |  |
| Red skin reaction |  |  | 1 | 2 | 3 | 4 |  |  |  |
| Retching as if to vomit but with no production |  |  | 1 | 2 | 3 | 4 |  |  |  |
| Skin colour changes |  |  | 1 | 2 | 3 | 4 |  |  |  |
| Shaking or tremors |  |  | 1 | 2 | 3 | 4 |  |  |  |
| Speech difficulties |  |  | 1 | 2 | 3 | 4 |  |  |  |
| Stomach cramps |  |  | 1 | 2 | 3 | 4 |  |  |  |
| Sweating after a meal |  |  | 1 | 2 | 3 | 4 |  |  |  |
| Swelling of the lower leg, feet or ankles |  |  | 1 | 2 | 3 | 4 |  |  |  |
| Urgency when needing the toilet |  |  | 1 | 2 | 3 | 4 |  |  |  |
| Visual difficulties |  |  | 1 | 2 | 3 | 4 |  |  |  |
| Weakness in lower limbs |  |  | 1 | 2 | 3 | 4 |  |  |  |
| Wound healing problems |  |  | 1 | 2 | 3 | 4 |  |  |  |

### Explanation of ratings

1. Can you explain why or why not the questions / concerns are relevant?

### Further evaluation of the EORTC QLQ-STO22 and list of concerns

1. Were there any questions / concerns that you think would be confusing / difficult to answer?
2. Can you explain what was confusing or difficult about the concerns / questions?
3. Were there any questions or concerns that you think might be upsetting?
4. Can you explain what was upsetting about these concerns / questions?

Some people we spoke to also suggested a few of the questions were confusing. Looking at the questions below; do you find these questions confusing? Would you prefer to see any of the alternative ways of asking the question?

**Question confusing? Yes No**

| Q37. Did you have a bloated feeling in your abdomen? |  |  |
| --- | --- | --- |

**Prefer the alternative? Yes No**

| Q37 alternative. Is retaining gas a problem for you? |  |  |
| --- | --- | --- |

**Question confusing? Yes No**

| Q40. Have you had trouble with belching? |  |  |
| --- | --- | --- |

**Prefer the alternative? Yes No**

| Q40 alternative. Is belching a problem for you? |  |  |
| --- | --- | --- |

**Do you think there is a better way of asking the question above?**

**Please detail:**

**Question confusing? Yes No**

| Q31  Have you had problems eating solid food? (Unsure if this means during or after eating?) |  |  |
| --- | --- | --- |

**Do you think there is a better way of asking the question above?**

**Please detail:**

**Question confusing? Yes No**

| Q46. Have you had trouble with eating in front of people? (doesn’t understand ‘trouble with eating’) |  |  |
| --- | --- | --- |

**Do you think there is a better way of asking the question above?**

**Please detail:**

Some people we spoke to also suggested a few of the questions were upsetting. Looking at the questions below; do you find these questions upsetting? Would you prefer to see any of the alternative ways of asking the question?

**Question upsetting? Yes No**

| Q49. Have you felt physically less attractive as a result of your disease or treatment? |  |  |
| --- | --- | --- |

**Do you think there is a better way of asking the question above?**

**Please detail:**

1. Are there any issues missing from the questionnaire or the list of concerns?

### Priority ratings (EORTC QLQ-C30 questions and list of concerns)

1. I would like you to think about the questions in this questionnaire *(EORTC QLQ-STO22)* and the list of concerns and think about which ones are the top priority questions or concerns to include in a quality of life questionnaire relevant for patients with gastric cancer.

### Summary and closure

1. Is there anything we have not spoken about regarding gastric cancer and its treatment which we have not covered?
2. Do you have any further comments about the questionnaire?
3. Thank you for your time and for sharing your experiences. If you would like to find out more about the study and the results, you are more than welcome to look on the EORTC Quality of Life Group *(remind participant of address on Participant Information Sheet).*

**Online resource 3**

Supplementary Table 1. Socio-demographic and clinical characteristics of patients (N=61) interviewed in Phase 1a

| Attribute |  | Total  (N=61) | East Asia (N=25) | Non-East Asia (N=36) |
| --- | --- | --- | --- | --- |
| Country | Cyprus | 5 |  | 5 |
|  | Japan | 20 | 20 |  |
|  | Malaysia | 5 |  | 5 |
|  | Mongolia | 5 | 5 |  |
|  | Spain | 5 |  | 5 |
|  | Turkey | 11 |  | 11 |
|  | UK | 10 |  | 10 |
| Age (years) | Mean  Range | 57.54  41-86 | 56.91  34-82 | 60.44  44-84 |
| Gender | Male | 31 | 10 | 21 |
|  | Female | 30 | 15 | 15 |
| Education level | University level or above | 25 | 11 | 14 |
|  | Post-compulsory school education | 17 | 5 | 12 |
|  | Compulsory school education | 13 | 4 | 9 |
|  | Less than school education | 0 | 0 | 0 |
|  | *Prefer not to answer / missing* | 5 | 4 | 1 |
| Occupation | Unskilled | 9 | 5 | 4 |
|  | Skilled manual | 18 | 8 | 10 |
|  | Administrative / middle management | 10 | 3 | 7 |
|  | Professional / senior management | 11 | 4 | 7 |
|  | *Prefer not to answer / missing* | 13 | 5 | 8 |
| Marital status | Married / partner | 43 | 16 | 27 |
|  | Separated / divorced / widowed | 11 | 3 | 8 |
|  | Single | 2 | 2 | 0 |
|  | *Prefer not to answer / missing* | 5 | 4 | 1 |
| Living arrangements | With family / loved ones | 52 | 20 | 32 |
|  | Alone | 4 | 2 | 2 |
|  | *Prefer not to answer / missing* | 5 | 4 | 1 |
| Ethnicity | Asian | 29 | 21 | 8 |
|  | White | 27 | 0 | 27 |
|  | *Prefer not to answer / missing* | 5 | 4 | 1 |
| Disease stage | Metastatic | 21 | 7 | 14 |
|  | Localised | 22 | 10 | 12 |
|  | Locoregional | 17 | 8 | 9 |
|  | *Missing* | 1 | 0 | 1 |
| Recurrence | None | 45 | 17 | 28 |
|  | Locoregional | 2 | 1 | 1 |
|  | Distant | 13 | 6 | 7 |
|  | *Missing* | 1 | 1 | 0 |
| Treatment | Surgery or endoscopic resection only | 8 | 6 | 2 |
|  | Perioperative chemotherapy and surgery or surgery and adjuvant chemotherapy | 23 | 14 | 9 |
|  | Pre- or post-operative chemoradiotherapy | 10 | 0 | 10 |
|  | Systemic therapy including chemotherapy‎/ targeted therapy‎ | 16 | 4 | 12 |
|  | Palliative care / treatment with palliative intent | 4 | 1 | 3 |
| Surgical approach | Open | 29 | 8 | 21 |
|  | Laparoscopic | 7 | 7 | 0 |
|  | Robotic | 2 | 2 | 0 |
|  | *Missing* | 5 | 0 | 5 |
| Surgery | Total gastrectomy | 21 | 9 | 12 |
|  | Distal gastrectomy | 9 | 9 | 0 |
|  | Proximal gastrectomy | 2 | 2 | 0 |
|  | Non-resectional surgery | 1 | 1 | 0 |
|  | *Missing* | 7 | 0 | 7 |
| Radiotherapy | Intensity-Modulated Radiation Therapy (IMRT) | 7 | 0 | 7 |
|  | Conformal Radiation Therapy (CRT) | 3 | 0 | 3 |
|  | *Missing* | 1 | 0 | 1 |
| Chemotherapy | FOLFOX (Oxaliplatin, Fluorouracil, Folinic acid) | 11 | 5 | 6 |
|  | Paclitaxel | 7 | 4 | 3 |
|  | Oxaliplatin | 5 | 5 | 0 |
|  | Paclitaxel and FOLFOX | 1 | 1 | 0 |
|  | Oxaliplatin and Capecitabine | 10 | 1 | 9 |
|  | Cisplatin | 1 | 1 | 0 |
|  | Docetaxel, Cisplatin, and S-1 | 1 | 1 | 0 |
|  | S-1 | 9 | 9 | 0 |
|  | FLOT (Fluorouracil, Folinic acid, oxaliplatin and Docetaxel) | 13 | 0 | 13 |
|  | FOLFIRI (Irinotecan, Fluorouracil and Folinic acid | 2 | 0 | 2 |
|  | Capecitabine and Cisplatin | 1 | 0 | 1 |
|  | Fluorouracil, Capecitabine, Oxaliplatin, Docetaxel | 1 | 0 | 1 |
|  | Epirubicin, Capecitabine, Oxaliplatin, Docetaxel | 1 | 0 | 1 |
|  | Fluorouracil | 2 | 0 | 2 |
|  | *Missing* | 2 | 0 | 2 |
| Targeted therapy | Nivolumab | 7 | 4 | 3 |
|  | Pembrolizumab | 3 | 0 | 3 |
|  | Ramucirumab | 5 | 3 | 2 |
|  | Trastuzumab | 4 | 0 | 4 |
| Co-morbidities | None | 34 | 17 | 17 |
|  | Diabetes | 9 | 2 | 7 |
|  | Hypertension | 9 | 3 | 6 |
|  | Cardiac | 4 | 1 | 3 |
|  | Rheumatism | 4 | 0 | 4 |
|  | Depression or anxiety | 2 | 1 | 1 |
|  | Stomach ulcers | 3 | 0 | 3 |
|  | *Missing* | 2 | 2 | 0 |
| ECOG Performance Status | Grade 0:  Fully active | 29 | 17 | 12 |
|  | Grade 1:  Restricted in physically strenuous activity | 20 | 6 | 14 |
|  | Grade 2:  Ambulatory and capable of all self-care | 10 | 1 | 9 |
|  | Grade 3:  Capable of only limited self-care | 1 | 0 | 1 |
|  | Grade 4:  Completely disabled | 0 | 0 | 0 |
|  | *Missing* | 1 | 0 | 1 |

**Online resource 4**

Supplementary Table 2. Socio-demographic and clinical characteristics of patients interviewed in Phase 1b

| Attribute |  | Total  (N=43) | East Asia (N=23) | Non-East Asia (N=20) |
| --- | --- | --- | --- | --- |
| Country | China | 5 | 5 |  |
|  | India | 3 |  | 3 |
|  | Japan | 8 | 8 |  |
|  | Mongolia | 5 | 5 |  |
|  | South Korea | 5 | 5 |  |
|  | Spain | 3 |  | 3 |
|  | Turkey | 5 |  | 5 |
|  | UK | 9 |  | 9 |
| Age (years) | Mean  Range | 60.29  31 - 84 | 60.86  40 - 84 | 59.63  31-83 |
| Gender | Male | 28 | 16 | 12 |
|  | Female | 15 | 7 | 8 |
| Education level | University level or above | 3 | 1 | 2 |
|  | Post-compulsory school education | 11 | 5 | 6 |
|  | Compulsory school education | 10 | 5 | 5 |
|  | Less than school education | 4 | 2 | 2 |
|  | *Prefer not to answer / missing* | 15 | 10 | 5 |
| Occupation | Unskilled | 8 | 6 | 2 |
|  | Skilled manual | 11 | 7 | 4 |
|  | Administrative / middle management | 3 | 0 | 3 |
|  | Professional / senior management | 5 | 0 | 5 |
|  | *Prefer not to answer / missing* | 16 | 10 | 6 |
| Marital status | Married / partner | 24 | 12 | 12 |
|  | Separated / divorced / widowed | 5 | 2 | 3 |
|  | Single | 0 | 0 | 0 |
|  | *Prefer not to answer / missing* | 14 | 9 | 5 |
| Living arrangements | With family / loved ones | 24 | 12 | 12 |
|  | Alone | 5 | 2 | 3 |
|  | *Prefer not to answer / missing* | 14 | 9 | 5 |
| Ethnicity | Asian | 17 | 9 | 8 |
|  | White | 12 | 0 | 12 |
|  | *Prefer not to answer / missing* | 14 | 9 | 5 |
| Disease stage | Metastatic | 19 | 8 | 11 |
|  | Localised | 15 | 8 | 7 |
|  | Locoregional | 9 | 7 | 2 |
| Recurrence | None | 38 | 19 | 19 |
|  | Locoregional | 0 | 0 | 0 |
|  | Distant | 5 | 4 | 1 |
| Treatment | Surgery or endoscopic resection only | 13 | 10 | 3 |
|  | Perioperative chemotherapy and surgery or surgery and adjuvant chemotherapy | 10 | 7 | 3 |
|  | Pre- or post-operative chemoradiotherapy | 4 | 1 | 3 |
|  | Systemic therapy including chemotherapy‎ or targeted therapy‎ | 14 | 3 | 11 |
|  | Palliative care / treatment with palliative intent | 2 | 0 | 2 |
| Surgical approach | Open | 10 | 6 | 4 |
|  | Laparoscopic | 15 | 11 | 4 |
|  | Robotic | 2 | 2 | 0 |
| Surgery | Total gastrectomy | 17 | 11 | 6 |
|  | Distal gastrectomy | 7 | 7 | 0 |
|  | Proximal gastrectomy | 2 | 1 | 1 |
|  | Non-resectional surgery | 1 | 0 | 1 |
| Radiotherapy | Conformal Radiation Therapy (CRT) | 2 | 0 | 2 |
|  | *Missing* | 1 | 0 | 1 |
| Chemotherapy | FOLFOX (Oxaliplatin, Fluorouracil, Folinic acid) | 2 | 0 | 2 |
|  | Oxaliplatin and Capecitabine | 4 | 0 | 4 |
|  | Cisplatin and Trastuzumab | 1 | 0 | 1 |
|  | FLOT (Fluorouracil, Folinic acid, Oxaliplatin and Docetaxel) | 2 | 0 | 2 |
|  | Fluorouracil + Oxaliplatin + Docetaxel | 3 | 0 | 3 |
|  | Capecitabine + Oxaliplatin + Cisplatin | 1 | 0 | 1 |
|  | Paclitaxel + FOLFOX | 1 | 1 | 0 |
|  | S-1+Docetaxel | 1 | 1 | 0 |
|  | SOX (S-1, Oxaliplatin) | 5 | 5 | 0 |
|  | LV5FU2 (Fluorouracil, Folinic acid) | 1 | 1 | 0 |
|  | SOX + Ramucirumab | 1 | 1 | 0 |
|  | *Missing* | 4 | 0 | 4 |
| Targeted therapy | Nivolumab | 1 | 0 | 1 |
|  | Pembrolizumab | 1 | 0 | 1 |
|  | Ramucirumab | 1 | 1 | 0 |
|  | Trastuzumab | 1 | 0 | 1 |
| Co-morbidities | None | 20 | 12 | 8 |
|  | AIDS/HIV | 1 | 1 | 0 |
|  | Hypertension | 7 | 5 | 2 |
|  | Diabetes | 6 | 1 | 5 |
|  | Cardiac | 4 | 1 | 3 |
|  | Depression or anxiety | 2 | 0 | 2 |
|  | Renal disease | 1 | 1 | 0 |
|  | Osteoarthritis | 1 | 0 | 1 |
|  | Hepatic disease | 1 | 0 | 1 |
|  | Missing | 1 | 1 | 0 |
| ECOG Performance Status | Grade 0:  Fully active | 12 | 9 | 3 |
|  | Grade 1:  Restricted in physically strenuous activity | 15 | 4 | 11 |
|  | Grade 2:  Ambulatory and capable of all self-care | 10 | 8 | 2 |
|  | Grade 3:  Capable of only limited self-care | 3 | 2 | 1 |
|  | Grade 4:  Completely disabled | 0 | 0 | 0 |
|  | *Missing* | 3 | 0 | 3 |
